# Suturing methods in prolapse surgery: a biomechanical analysis

**DOI:** 10.1007/s00192-020-04609-6

**Published:** 2020-12-02

**Authors:** J. Hachenberg, A. Sauerwald, H. Brunke, S. Ludwig, M. Scaal, A. Prescher, C. Eichler

**Affiliations:** 1 Department of Gynecology and Obstetrics, Hannover Medical School, Hannover, Germany; 2 Department of Gynecology and Obstetrics, St. Marien Hospital Düren, Düren, Germany; 3 Department of Gynecology and Obstetrics, University of Cologne, Cologne, Germany; 4 Department of Anatomy, RWTH Aachen University, Aachen, Germany; 5 Department of Gynecology and Obstetrics, Frauenklinik Holweide, Kliniken der Stadt Köln, Cologne, Germany; 6 Department of Anatomy II, University of Cologne, Cologne, Germany; 7 DZMGS (German Center for Material Science in Gynecology and Senology), Cologne, Germany

**Keywords:** Pelvic organ prolapse, Uro-gynecological surgery, Biomechanical testing, Suturing technique, Laparoscopy

## Abstract

**Introduction:**

Pelvic organ prolapse is a common problem in urogynecological surgery. Abdominal and laparoscopic sacrocolpopexy is currently considered to be the gold standard of treatment. The main problem remains the anatomical point of fixation as well as how sutures are placed. We evaluated the biomechanical difference between an in-line ligament suture versus an orthogonal ligament suture and a single suture versus a continuous suture at the anterior longitudinal ligament in an in-vitro, sacrocolpopexy model.

**Methods:**

Biomechanical in-vitro testing was performed on human, non-embalmed, female cadaver pelvises. An Instron test frame (tensinometer) was used for load/ displacement analysis. The average patient age was 75 years. Ligament preparation yielded 15 ligaments available for testing. Recorded parameters were the ultimate load, failure displacement, and stiffness.

**Results:**

This in-vitro analysis of different suturing methods showed the difference between an orthogonal and an in-line approach to be the ultimate load. Orthogonal sutures displayed an ultimate load of 80 N while in-line suturing yielded only 57 N (*p* < 0.05). For the anterior longitudinal ligament, this study demonstrated that continuous suture is significantly superior to a single suture regarding failure displacement (*p* < 0.05).

**Conclusion:**

We established baseline biomechanical parameters for the sacrospinous ligament and anterior longitudinal ligament. An orthogonal suture is superior to an in-line suture in an in-vitro model. A continuous suture is superior to a single suture at the anterior longitudinal ligament. Clinical trials might be able to evaluate whether any clinical significance can be established from these findings.

## Introduction

Pelvic organ prolapse (POP) is a common reason for the admission of women to hospitals and indications for laparoscopic surgery. Uterine prolapse affects up to 14.2% of women [1]. Society as a whole is aging and life expectancy is increasing. Age and obesity are considered the main risk factors for POP. Therefore, the incidence of POP is likely to increase in the future with 11–19% of women undergoing POP surgery [2, 3]. In the US approximately 300.000 surgeries are performed each year to treat POP (https://www.fda.gov/downloads/MedicalDevices/Safety/AlertsandNotices/UCM262760.pdf). Abdominal sacrocolpopexy is currently considered to be the gold standard of treatment whereas laparoscopic prolapse surgery is becoming a widely used alternative. Optimizing surgical techniques will hopefully reduce operating time and therefore, patient morbidity.

Despite several innovative and different new developments in laparoscopy, including robotic surgery as well as standard laparoscopic procedures, a major problem remains regarding the point of fixation as well as the manner of suture placement [4–7]. In a previous biomechanical analysis, we evaluated the value of combining several sutures compared to a single suture with or without artificial mesh interpolation at the ileo-pectineal ligament [8]. Our results demonstrated that a single suture may be sufficient for adequate suspension. Further biochemical analysis is required to evaluate the choice of suspension ligament as well as suture placement to ligament fiber orientation.

Literature very adequately summarizes the historical value and individual benefits of transvaginal approaches such as Amreich-Richter or Sederl-Richter surgical methods [9]. In these cases, the sacrotuberous ligaments and later sacrospinous ligaments were used as a fixation point for prolapse suspension [10]. This method has been thoroughly evaluated and the fixation ligament i.e. the sacrospinous ligament may be considered as a baseline for testing [11]. Fairclough et al. collected data from 35 centers in the UK regarding key practice points for prolapse surgery. Despite over 50 years of modern prolapse surgery, the authors found that there is remarkably little evidence supporting the main steps of prolapse surgery [12].

The goal of this biomechanical analysis was to evaluate the main biomechanical parameters of the sacrospinous ligament and anterior longitudinal ligament to establish baseline values.

1. Establish a baseline reading for sacrospinous ligament biomechanical parameters for a single suture including: Maximum Load (N), Displacement and Failure (mm), and Stiffness (N/mm).
2. Establish a baseline reading for anterior longitudinal ligament biomechanical parameters for a single suture including: Maximum Load (N), Displacement and Failure (mm), and Stiffness (N/mm).
3. Compare in-line suturing with orthogonal suturing for the sacrospinous ligament.
4. Compare single suture with continuous suture approaches for the anterior longitudinal ligament.
5. Compare these findings to current literature.

## Methods

**Fig. 1:**
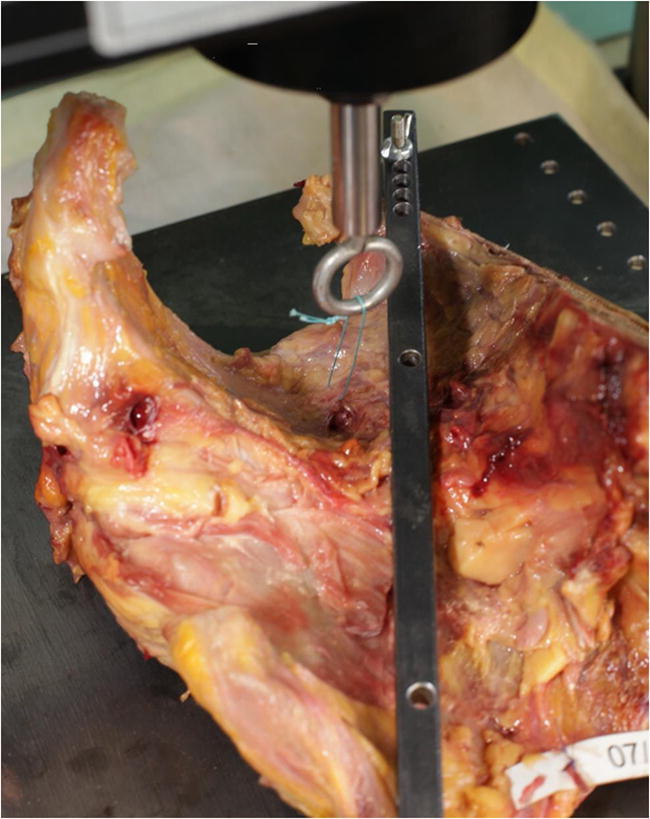
Instron 5565 test frame. A picture of the Instron 5565 test frame used in the study

The evaluation procedure for assessing biomechanical parameters in this study has been established in a previous publication [8]. Figure 1 shows the Instron 5565 test frame used in the study. Similar to this method we performed all experiments on human non-embalmed, fresh, female cadaver pelvises. As standard practice for anatomic studies at the institute of anatomy Anatomy of RWTH Aachen University, we used a formalin-based cadaver embalming technique to prepare the cadavers for anatomical studies. Anatomical preparation of the sacrospinous ligament was performed by an experienced gynecological surgeon. The average age of the patient was 75 years old and anatomical preparation yielded fifteen available ligaments obtained from a total of eight. One pelvis did not present a sacrospinous ligament. All cadavers were procured from the Institute of Anatomy at the University of Aachen. Identifying data was available to only one co-author, A. Prescher.

A total of fourteen trials were performed with the sacrospinous ligament and sixteen tests were conducted for the anterior longitudinal ligament. This resulted in a total of 30 test trials, all of which could be used for evaluation. The following groups were divided for analysis:

- Group 1 (*n* = 7) evaluated the sacrospinal ligament with an orthogonal suture (S O)
- Group 2 (n = 7) evaluated the sacrospinal ligament with an in-line suture (S IL)
- Group 3 (*n* = 8) evaluated a single suture placed orthogonally to the anterior longitudinal ligament (L O)
- Group 4 (n = 8) evaluated a continuous orthogonal suture running on the anterior longitudinal ligament (L C)

**Fig. 2:**
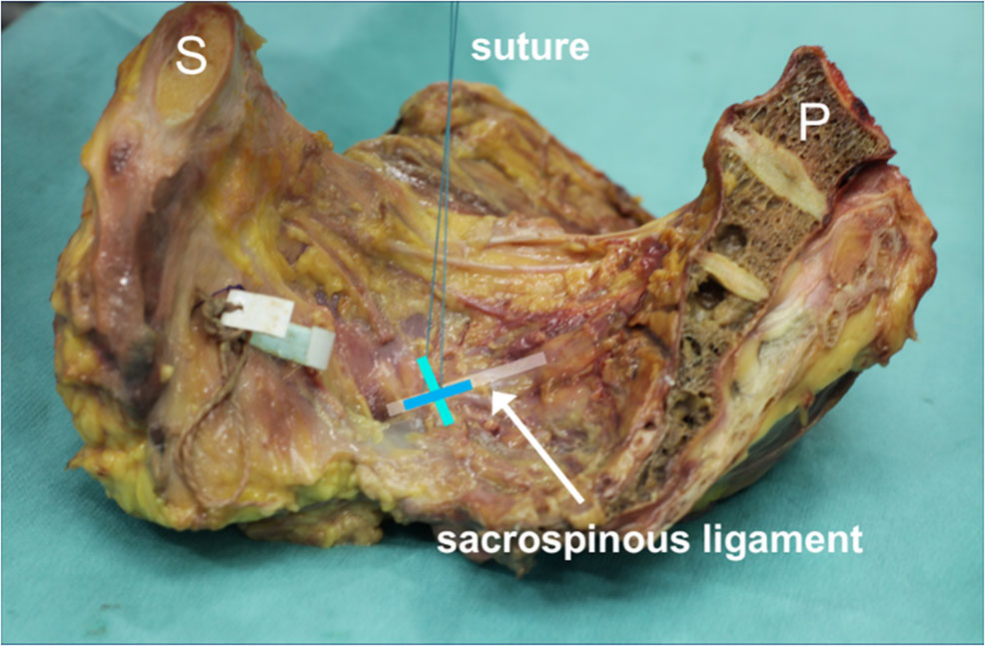
Testing Setup. This image shows the testing setup. Shown is an orthogonal suture attached to the sacrospinous ligament before the introduction into the test frame. (dark green = fiber direction; turquois = suture direction)

The first two groups evaluated suture placement, group three evaluated the potential ligament, and group four evaluated the suturing method. A synthetic, braided, non-absorbable Ethibond suture 0, FSLX needle, 75 cm green filament (Ethicon/ Johnson & Johnson, Somerville, NJ, USA) was used in all four groups. Figure 2 shows the sacrospinous fixation points. The analysis was performed on an Instron 5565® test frame (Fig. 1) using the Bluehill 2 Software®. All tests were transient evaluations of the individual fixation methods at 5 N/s load increase. Recorded parameters were the ultimate load (N) and failure displacement (mm). These resulted in calculated parameters such as stiffness (N/mm) and load at 2 mm displacement. The latter being considered as fixation failure in biomechanical evaluations since stability may be lost as dehiscence exceeds 2 mm [8, 13–17].

### Statistics

Statistics Statistical analysis was performed using the VassarStats1 (Vassar College, Poughkeepsie, NY, USA) statistics program. ANOVA analysis and t-tests were used to evaluate significances when appropriate.

### Ethics Committee Approval

This study was conducted under institutional review board standard operating procedures. An ethics committee vote was initiated, but deemed unnecessary by the “Ethikkommission der Aerztekammer Nordrhein”. A written statement to this extent is available.

## Results

A total of fourteen trials were performed with the sacrospinous ligament and sixteen were conducted for the anterior longitudinal ligament. This resulted in a total of 30 test trials that could be used for evaluation.

**Fig. 3:**
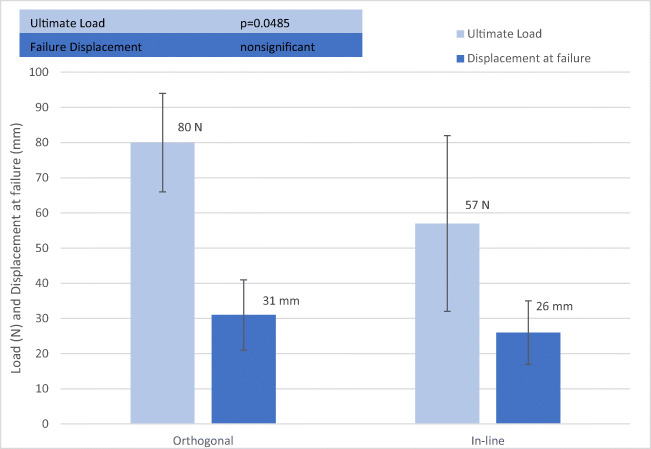
Comparison of Orthogonal Suture and In-Line Suture of Sacrospinous Ligament for Ultimate Load and Failure Displacement. Error bars represent standard deviations

A summary of orthogonal suture vs. in-line suture results is given in Table 1 including *p* values. Figures 5 and 6 illustrate the difference between suture placement techniques. Group 1 represents the orthogonal suture trial, whereas group 2 represents the in-line suture-trials (Table 1). Figure 3 shows the comparison between the two groups for Ultimate Load and Failure Displacement. The results show that the main difference between the „orthogonal “and the „in-line “approach is the ultimate load with a significant difference of 80 N to 57 N (*p* = 0.0485). For the parameters, displacement-at-failure, and stiffness no significant difference between the two techniques (*p* > 0.05) could be shown. Lastly, the load at 2 mm displacement was calculated as well and likewise did not show any significant differences.

**Table 1:** Comparison of orthogonal suture vs. In-Line suture of Sacrospinous Ligament

Evaluated Entity	n	Ultimate Load	Failure Displacement	Stiffness
Total Trials = 14		N	mm	N/mm
**Group 1 (Orthogonal Suture)**	7	80	31	3.30
**Group 2 (In-line Suture)**	7	57	26	3.17
***p value***		0.0485	>0.05	>0.05

All dynamic testing was completed; no global failures occurred; the steady state was reached in all cases

**Fig. 4:**
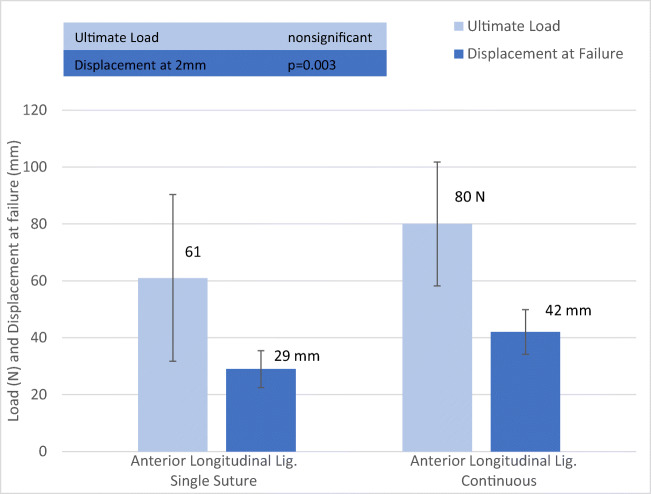
Comparison of Single Suture and Continous Suture of Anterior Longitudinal Ligament for Ultimate Load and Failure Displacement. Error bars represent standard deviations

**Fig. 5:**
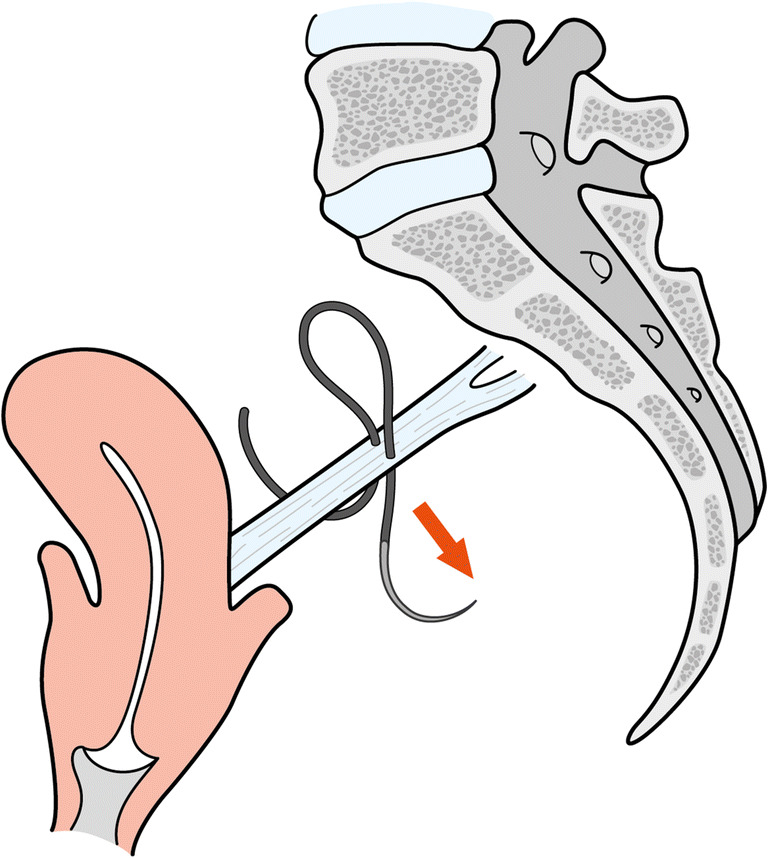
Orthogonal suture of sacrospinous ligament. Arrow represents the direction of suture placement

**Fig. 6:**
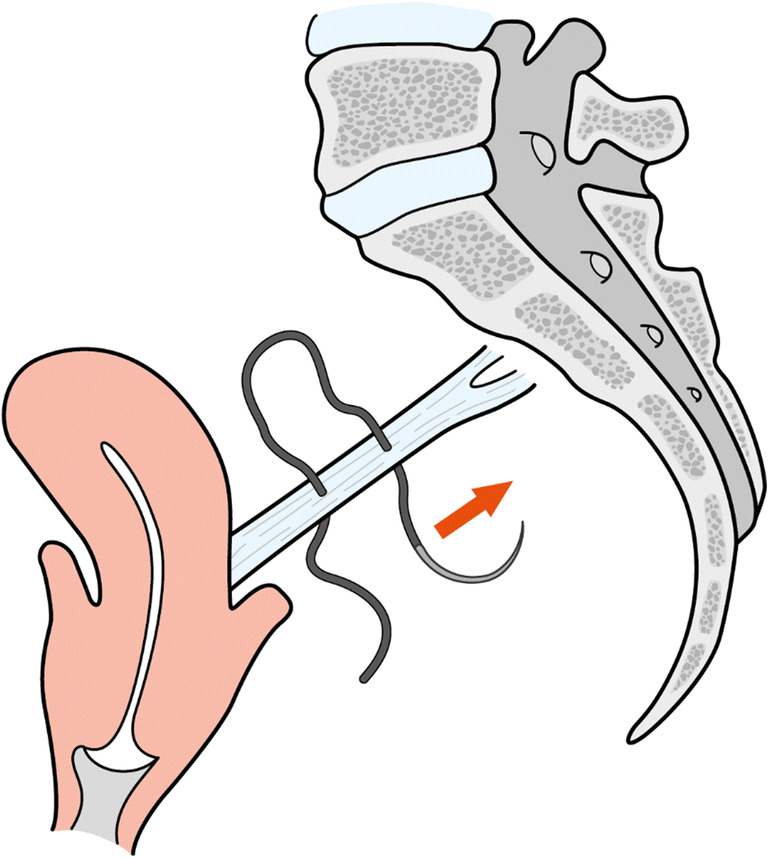
In-Line suture of sacrospinous ligament. Arrow represents the direction of suture placement

Figure 4 shows the single suture vs. continuous suture of the anterior longitudinal ligament. Group 1 represents the single suture trials of the anterior longitudinal ligament. Group 2 comprises the continuous suture trials (Table 2). Ultimate Load and Stiffness did not yield significantly different results (*p* > 0.05) whereas failure displacement was significantly higher for group 2 (*p* = 0.003). The calculated load at 2 mm displacement was calculated as well and did not show any significant differences (p > 0.05) (Figs. 5 and 6).

**Table 2:** Comparison of Single Suture and Continous Suture of Anterior Longitudinal Ligament

Evaluated Entity	n	Ultimate Load	Failure Displacement	Stiffness
Total Trials = 16		N	mm	N/mm
**Group 1 (Single Suture)**	8	61	29	2.99
**Group 2 (Continous Suture)**	8	80	42	2.52
***p value***		>0.05	0.003	>0.05

All dynamic testing was completed; no global failures occurred; steady-state was reached in all cases

## Discussion

In our study, we compared two laparoscopic suturing techniques for the sacrospinous ligament and the anterior longitudinal ligament. This work established a baseline of biomechanical parameters for both ligaments for a single suture in terms of: Maximum load (N), Failure displacement (mm), and Stiffness (N/mm). For the sacrospinous ligament, the data showed significant differences between the two suturing techniques in terms of the ultimate load. This means that an orthogonal suture can carry more load than an in-line suture. White et al. found that the same applies to the anterior longitudinal ligament. They studied the optimal suture placement in abdominal sacrocolpopexy by comparing different levels of suture placement. In the lower levels, one or two centimeters below the os sacrum, the horizontally placed sutures were stronger. [18]. An explanation for this can be found in the histological structure of the ligament itself. Neumann et al. investigated the properties of the anterior longitudinal ligament and found that the strength in direction of the length of the ligament is higher than the strength in direction of width [19]. Our results suggest that this is similarly true for the sacrospinous ligament though no histological evaluation is available. An orthogonal suture, therefore, uses the strength of the stronger part of the ligament resulting in a higher ultimate load. Thus, regardless of the type of ligament, it should, therefore, be the goal of any surgeon to avoid in-line suturing. Our results show a significant difference in ultimate load, favoring the orthogonal suturing technique, by a factor of 1.4 (*p* < 0.05). Results indicate that this ultimate load of an orthogonal suture is likely not translated into a stronger fixation as the failure displacement as well as stiffness did not show significant differences between the two techniques.

Where White et al. only described the optimal suturing placement in abdominal sacrocolpopexy, this study systematically offers a baseline evaluation of biomechanics of the sacrospinous ligament and the anterior longitudinal ligament. Comparing these findings to the current literature, there has thus far not been a study evaluating two endoscopic suture techniques by biomechanical features. This work is therefore the first of its kind. There is no data available on gynecological biomechanical analyses at all except for data provided by this study group. Little evidence through high-quality studies is provided for the main steps of sacrocolpopexy. This is valid for the evaluation of optimal suturing techniques as well. In 2015 Stavropoulos et al. evaluated different techniques of endoscopic suturing in gastrointestinal endoscopy by applying an Apollo OverStitch suturing device in a variety of different ways such as the closure of perforations, stent fixation, or fistula closure [20].

While there are a plethora of clinical studies comparing different surgical techniques, there is not one study that focuses on single suturing steps commonly used in multiple surgical techniques indicating that this field might require further investigation especially as to the biomechanical features of suturing techniques and suturing material. In this regard, there are two studies worth citing in gynecological surgery: Allahdin et al. compared the suturing material itself finding that Vicryl is overall the superior material [21]. Sauerwald and Eichler et al. conducted a study to compare single suture techniques versus continuous suturing techniques finding that there was no difference between the ultimate load of a single interrupted suture versus a continuous one for the ileo-pectineal ligament [8]. However, most interestingly in this study, we were able to show that for failure displacement a continuous suture in the anterior longitudinal ligament is superior to a single suture (Table 2). Similar to the findings of Sauerwald and Eichler there was no difference in the ultimate load and stiffness. Our study proves that the evaluation of biomechanical features of different fixation points is crucial. As stated prior, essential steps of sacrocolpopexy lack evidence. This study provides the greatly needed data for evidence-based optimization of sacrocolpopexy. Clinical trials might be effective in evaluating whether any clinical significance can be established from these findings.

## Conclusion

We established baseline biomechanical features for the sacrospinous ligament and anterior longitudinal ligament. Our results show that an orthogonal suture is superior to an in-line suture in an in vitro setting. Orthogonal sutures should be preferred to in-line suture whenever possible. A continuous suture is superior to a single suture at the anterior longitudinal ligament. Clinical trials might be able to evaluate whether a clinical significance can be established from these findings.
